# HIV associated psychiatric comorbidity among attendees at a tertiary hospital, North-Eastern Nigeria

**DOI:** 10.4102/sajpsychiatry.v29i0.2022

**Published:** 2023-07-31

**Authors:** Akinbola R. Oyedun, Ganiyu O. Oluwatoyin

**Affiliations:** 1 Department of Psychiatry, Faculty of Clinical Sciences, Gombe State University, Gombe, Nigeria; 2 Department of Medicine, Federal Teaching Hospital Gombe, Gombe, Nigeria; 3 Department of Medicine, Moddibo Adamawa University Teaching Hospital, Yola, Nigeria

**Keywords:** HIV, AIDS psychiatric comorbidity, stigma, depression, psychosis, psychopathology, comorbidity, PLWHA

## Abstract

**Background:**

There are a wide range of neuropsychiatric conditions associated with human immunodeficiency virus (HIV) and acquired immunodeficiency syndrome (AIDS). These mental disorders may be unrecognised yet their presence can significantly affect outcome.

**Aim:**

This study aimed to determine psychiatric comorbidity associated with HIV and AIDS.

**Setting:**

The HIV clinic of a tertiary hospital in North-Eastern Nigeria.

**Methods:**

A cross-sectional descriptive study consecutively recruiting 328 adult persons living with HIV. The Mini International Neuropsychiatric Interview and a sociodemographic questionnaire were administered to the participants.

**Results:**

Two-thirds of the respondents were females. The mean age (±s.d.) was 42 years (±11.24). Majority of the participants had World Health Organization stage 1 HIV disease. The prevalence of psychiatry comorbidity among our respondents was 82.9%. Social phobia was the leading disorder (69.8%). Others were mixed depression anxiety disorder (49.4%) and post-traumatic stress disorder (36.6%). Current psychosis was 27.7%, while major depressive disorder was 12.2%. Psychiatric comorbidity was significantly associated with male gender, religion, ethnicity, marital status and being unemployed with *p* < 0.01. Human immunodeficiency virus stage was related to panic disorder with *p* < 0.01, while viral load was significantly associated with depressive disorder with *p* = 0.001.

**Conclusion:**

Majority of our HIV patients attending the clinic have undetected psychiatric morbidity. Clinicians need to be aware of the features of major psychiatric disorders and refer appropriately for improved overall outcome.

**Contribution:**

This study contributes to the body of work on unrecognised psychiatric comorbidity in people living with HIV and AIDS, especially in North-Eastern Nigeria, identifying issues which are relevant to clinical practice and buttressing the need for integration of mental healthcare services into HIV treatment and prevention services.

## Introduction

People living with human immunodeficiency virus (HIV) and acquired immunodeficiency syndrome (AIDS) (PLWHA) are more likely to have a higher population with undiagnosed mental illness than the general population.^1,2^ Psychiatric disorders in this group of persons often go undetected as they are not the focus of treatment. However, it leads to substantially increased morbidity^1^ and even mortality.^3^

A systematic review of HIV positive adults in Africa showed that about one-half of HIV positive adults have some form of psychiatric disorder.^4^ A study from East Africa showed that despite a prevalence of psychiatric comorbidity of 46.5% in the respondents, only 3.6% had a previously diagnosed mental illness.^5^ Nigerian studies show that the prevalence of both of anxiety and depression can be as high as 48% in PLWHA,^6^ with another Nigerian study reporting that two-fifths of respondents had various forms of psychiatric comorbidity including substance use, depression and anxiety.^7^

There is a bidirectional relationship between a positive HIV status and mental health disorders,^6,8,9^ with neuroinflammatory and certain stress-related factors playing contributory roles.^3^ On the one hand, having a preexisting mental health disorder might be a contributory factor to contracting the virus, through engaging in risky behaviour such as injection drug use or engaging in risky sexual behaviour in those who have substance use disorders.^3^ This increased risk of contracting HIV has been reported among individuals with substance use disorder,^9^ while Wandel et al. showed that the prevalence of substance use disorders was high in HIV positive persons even before the diagnosis of HIV.^10^ Risky behaviour can also occur in those who are disinhibited in certain affective disorders like mania.^11^ On the other hand, having a chronic illness, such as HIV, can predispose to mental health disorders both from the development of AIDS and HIV-associated neurocognitive disorder (HAND)^10^ as well as from various psychological and social processes such as stigma.^12^ In addition, antiretrovirals,^13^ viral load^1^ and decrease in functioning^1^ can predispose to psychiatric morbidity.

Several mental health conditions are associated with HIV.^1^ Anxiety disorders are prevalent, with an analysis of global prevalence of anxiety disorders showing a prevalence of 22.85%, almost 5% higher than that in the general population.^14^ Social phobia has been found in PLWHA, with older quantitative studies reporting up to 14.8% of PLWHA have social phobia^15^ and a study in North-Central Nigeria reporting a prevalence of 4.0%.^16^ Qualitative studies among women in Iran report that the root cause of social phobia is stigma.^17^ Stigma and other psychosocial stressors are linked to the neuroendocrine stress response. They potentiate residual immune dysregulation and alter other biobehavioural processes relevant to health outcomes.^9^

Suicide ideation is also common among PLWHA and it is very important to do a risk assessment.^2^ A systematic review and meta-regression analysis by Tsai et al. revealed a pooled prevalence estimate of suicidal ideation, attempted suicide and deaths by suicide of 22.3%, 9.6% and 1.7%, respectively.^18^ The most common psychiatric conditions predicting suicide are dysthymia, borderline personality and the substance use disorders.^19^

This study seeks to bridge the gap between research and practice in the zone where this study was carried out to show there is a need for mental health assessments in PLWHA when they come for follow-up, as early identification and management can improve overall outcomes.

### Aim

This study aimed to determine if there is psychiatric comorbidity and its relationship with clinical correlates and sociodemographic variables among HIV and AIDS clinic attendees of a tertiary health facility. The specific objectives are:

- To determine the prevalence of psychiatric comorbidity among HIV clinic attendees.
- To determine the association between sociodemographic variables, clinical correlates and psychiatric comorbidity in PLWHA.

### Significance of the study

While there are a lot of studies on HIV and AIDS and also on psychiatric morbidity in Nigeria, there are fewer studies on HIV-associated psychiatric comorbidity and fewer still published studies in the immediate locale where this study took place. It is thus hoped that this study will not only help to bridge a data gap in research into HIV and AIDS, but also highlight their plight *vis-à-vis* mental health access and ensure that mental health is also prioritised for those living with this condition.

## Research methods and design

This was a cross-sectional descriptive study design.

### Setting

The study was carried out in North-Eastern Nigeria, in Gombe State, a multi-ethnic state with 3 million residents, who are predominantly of Fulani ethnicity.^20^ The community is strongly religious and all government-owned secondary healthcare centres in each local government have their own HIV units.

The tertiary hospital is a 300-bed specialist tertiary healthcare facility owned by the federal government of Nigeria running specialty clinics and consultations in various fields of medicine. It renders specialist care to the inhabitants of the state and neighbouring states. The HIV unit runs clinics daily, with a usual attendance of about 100 patients. Stable patients are generally seen every 3–6 months.

### Sampling

#### Inclusion criteria

Patients who were 18 years and older with confirmatory HIV test results and who consented to take part in the study were included.

#### Exclusion criteria

Patients who did not understand English or Hausa language or those with severe physical or mental conditions who would be unable to fill the questionnaire were excluded from the study.

### Sample size calculation

Sample size was estimated by:


$$n=z^{2}pq/d^{2}$$ Eqn 1

where *z* = standard deviation set at 1.96 (this corresponds to 95% confidence interval), *p* = the proportion in the target population estimated to have the desired characteristic and was set at 71.4% (i.e. 0.714),^1^ *q* = 1.0 – *p* and *d* = level of precision (0.05) since degree of precision desired is 95%. Therefore, *n* = (1.962)^2^*0.714*0.286/(0.052) *n* = 314. (However, the data were eventually collected from 328 respondents.)

### Instruments and data collection procedure

To facilitate data collection, structured and semi-structured instruments were used. The Mini International Neuropsychiatric Interview (MINI) Plus which can screen and diagnose a wide range of psychiatric disorders was used to assess psychiatric comorbidity. This instrument can also make a ‘current’ (in the last 1 month) diagnosis as well as a ‘lifetime’ (anytime in the past) diagnosis and has been used extensively in similar studies,^1,5^ as well as a sociodemographic questionnaire.

Ten booked clinic attendees were recruited per day through a random selection method, provided they fulfilled the inclusion criteria and they gave consent. The nature of the study was explained to the selected patients to obtain their consent for the study. Respondents were then administered the MINI Plus instrument as well as a sociodemographic questionnaire. Information needed was asked directly from patients and medical diagnosis and comorbidity was obtained from the case file. The managing physicians filled the clinical characteristics for each patient.

### Ethical considerations

Ethical approval to carry out the study was obtained from the Health Research Ethics committee of the hospital: approval code NHREC/25/10/2013. Consent was read out to them from the consent form, and oral permission was obtained from each patient before inclusion in the study. The decision to participate in the study was voluntary and they were free to withdraw consent. Consent and confidentiality of participants was ensured as they filled the questionnaires. Participants were assured of anonymity and confidentiality even during dissemination of findings. However, participants identified with mental or behavioural disorders were referred for formal psychiatric evaluation and management.

### Data analysis

Study data were analysed using Statistical Package for Social Sciences (SPSS) programme, version 21.0. Descriptive statistics were presented using frequency distribution tables reflecting percentages, mean and standard deviation. Medians were preferred as the measure of central tendency for data that were skewed. Between-group differences in categorical variables, such as gender, religion, place of residence, etc., were compared using chi-square statistics, while differences in continuous variables, such as age, were compared using independent students *t*-test. The confidence interval set at 95% and *p-*value** less than 0.05 (*p* < 0.05) were taken to be significant. The following disorders were used to model ‘psychiatric comorbidity’: (1) current depressive disorder, (2) current hypomania or mania, (3) current psychotic symptoms, (4) social anxiety disorder, (5) obsessive compulsive disorder, (6) generalised anxiety disorder, (7) somatisation, (8) hypochondriasis, (9) adjustment disorder, (10) mixed depression anxiety disorder, (11) panic disorder and (12) post-traumatic stress disorder (PTSD).

## Results

The mean age (± s.d.) of participants was 41.89 years (± 11.24) and it ranged between 18 and 71 years (Table 1).

**TABLE 1:** Sociodemographic variables (*N* = 328).

Sociodemographic characteristics of respondents	Total	%
**Gender**		
Male	108	32.9
Female	220	67.1
Total	328	100.0
**Age groups (years)**		
Young	133	40.5
Middle aged	174	53.0
Elderly	21	6.4
Total	328	100.0
**Ethnicity**		
Hausa	201	61.3
Fulani	61	18.6
Other tribes	66	20.1
Total	328	100.0
**Religion**		
Christian	153	46.6
Muslim	175	53.4
Total	328	100.0
**Place of abode**		
State capital	244	74.4
Within the state	60	18.3
Outside the state	24	7.3
Total	328	100.0
**Marital status**		
Single	55	16.8
Married	205	62.5
Separated	29	8.8
Widowed	39	11.9
Total	328	100.0
**Living circumstance**		
Alone	25	7.6
With nuclear	271	82.6
Other family	29	8.8
Non-family	3	0.9
Total	328	100.0
**Employment status**		
Employed	133	40.5
Unemployed	176	53.7
Retired	19	5.8
Total	328	100.0

### Psychiatric comorbidity and its association with sociodemographic variables

Psychiatric comorbidity was found in 272 (82.9%) participants as assessed by the MINI (Table 2) with significant associations between psychiatric comorbidity and gender, ethnicity, religion, marital status and employment status.

**TABLE 2:** Relationship between sociodemographic features and psychiatric comorbidity.

Sociodemographic characteristics of respondents	Psychiatric comorbidity						χ^2^	*P*
	Absent (*N* = 56)		Present (*N* = 272)		Total (*N* = 328)			
	*n*	%	*n*	%	*n*	%		
**Gender**							13.03	**< 0.01**
Male	30	27.8	78	72.2	108	32.9	-	-
Female	26	11.8	194	88.2	220	67.1	-	-
Total	56	17.1	272	82.9	**328**	100.0	-	-
**Age groups (years)**							5.63†	0.05
Young	17	12.8	116	87.2	133	40.5	-	-
Middle aged	32	18.4	142	81.6	174	53.0	-	-
Elderly	7	33.3	14	66.7	21	6.4	-	-
Total	56	17.1	272	82.9	328	100.0	-	-
**Ethnicity**							26.00	< 0.01**
Hausa people	26	12.9	175	87.1	201	61.3	-	-
Fulani people	5	8.2	56	91.8	61	18.6	-	-
Other tribes	25	37.9	41	62.1	66	20.1	-	-
Total	56	17.1	272	82.9	328	100.0	-	-
**Religion**							10.24	< 0.01**
Christian	37	24.2	116	75.8	153	46.6	-	-
Muslim	19	10.9	156	89.1	175	53.4	-	-
Total	56	17.1	272	82.9	328	100.0	-	-
**Place of abode**							6.24	0.06
State capital	47	19.3	197	80.7	244	74.4	-	-
Within the state	4	6.7	56	93.3	60	18.3	-	-
Outside the state	5	20.8	19	79.2	24	7.3	-	-
Total	56	17.1	272	82.9	328	100.0	-	-
**Marital status**							17.76†	< 0.01**
Single	12	21.8	43	78.2	55	16.8	-	-
Married	43	21.0	162	79.0	205	62.5	-	-
Separated	0	0.0	29	100.0	29	8.8	-	-
Widowed	1	2.6	38	97.4	39	11.9	-	-
Total	56	17.1	272	82.9	328	100.0	-	-
**Living circumstance**								
Alone	4	16.0	21	84.0	25	7.6	-	-
With nuclear	47	17.3	224	82.7	271	82.6	-	-
Other family	5	17.2	24	82.8	29	8.8	-	-
Non-family	0	0.0	3	100.0	3	0.9	-	-
Total	56	17.1	272	82.9	328	100.0	-	-
**Employment status**							18.79†	< 0.01**
Employed	33	24.8	100	75.2	133	40.5	-	-
Unemployed	16	9.1	160	90.9	176	53.7	-	-
Retired	7	36.8	12	63.2	19	5.8	-	-
Total	56	17.1	272	82.9	328	100.0	-	-

† Expected cell count < 5 Fisher’s exact test used.

* , *p* < 0.05;

** , *p* < 0.01.

### Prevalence of individual psychopathology or psychiatric diagnosis

Table 3 shows the most common disorder was social phobia 229 (69.8%). Mixed depression anxiety disorder 162 (49.4%) followed by Post traumatic stress disorder 120 (36.6%). Sixty respondents had suicide ideation.

**TABLE 3:** Prevalence of individual psychiatric comorbidity (*N* = 328).

Clinical condition or psychopathology	Absent		Present	
	*n*	%	*n*	%
Lifetime psychosis	207	63.1	121	36.9
Current psychosis	237	72.3	91	27.7
Depressive disorder lifetime	272	82.9	56	17.1
Depressive disorder (current)	288	87.8	40	12.2
Suicide ideation	268	81.7	60	18.3
Hypomania or mania	273	83.2	55	16.8
Mixed depression anxiety	166	50.6	162	49.4
Social phobia	99	30.2	229	69.8
Obsessive compulsive disorder	284	86.6	44	13.4
Generalised anxiety disorder	254	77.4	74	22.6
Somatisation disorder	304	92.7	24	7.3
Unspecified anxiety disorders	297	90.5	31	9.5
Hypochondriasis	278	84.8	50	15.2
Adjustment disorder	289	88.1	39	11.9
Panic disorders	321	97.9	7	2.1
Post-traumatic stress disorder	208	63.4	120	36.6
Alcohol use disorders	299	91.2	29	8.8
Substance use disorders	321	97.9	7	2.1

### Alcohol and drug use

Lifetime alcohol use was significantly related to developing psychiatric comorbidity. Current cigarette smoking or current use of psychoactive substances was unrelated to psychiatric comorbidity (Table 4).

**TABLE 4:** Alcohol and drug use.

Psychoactive substance	Psychiatric comorbidity						χ^2^	*P*
	Absent		Present		Total (*N* = 328)			
	*n*	%	*n*	%	*n*	%		
**Lifetime use of alcohol**							4.40	0.04*
No	39	14.9	223	85.1	262	79.9	-	-
Yes	17	25.8	49	74.2	66	20.1	-	-
Total	56	17.1	272	82.9	328	100.0	-	-
**Current use of alcohol**							2.37	0.12
No	47	16.0	247	84.0	294	89.6	-	-
Yes	9	26.5	25	73.5	34	10.4	-	-
Total	56	17.1	272	82.9	328	100.0	-	-
**Lifetime cigarette smoking**							2.48	0.12
No	48	16.1	251	83.9	299	91.2	-	-
Yes	8	27.6	21	72.4	29	8.8	-	-
Total	56	17.1	272	82.9	328	100.0	-	-
**Current cigarette smoking**							1.02	0.31
No	52	16.6	261	83.4	313	95.4	-	-
Yes	4	26.7	11	73.3	15	4.6	-	-
Total	56	17.1	272	82.9	328	100.0	-	-
**Current use of illicit drugs**							0.06	0.80
No	54	17.0	264	83.0	318	97.0	-	-
Yes	2	20.0	8	80.0	10	3.0	-	-
Total	56	17.1	272	82.9	328	100.0	-	-

### Clinical parameters

All respondents had commenced antiretroviral (ARV) medication, 14 (4.1%) of respondents had commenced antiretroviral medication in the last year and 14 (4.1%) were not currently using cotrimoxazole. The most common medication was combination of tenofovir, lamivudine and dolutegravir (TLD) at 89%. Nine-tenths of them were on first-line medication.

Body mass index (BMI) ranged from 10.6 to 46.9 with a mean of 23.7 (±5.37). Nineteen (5.6%) of them were hypertensive, 8 (2.35%) had a current febrile illness and 1 person had confirmed tuberculosis.

Only BMI was significantly related to psychiatric comorbidity (Table 5), and when multivariate analysis was performed, the likelihood ratio test value was 0.048 with goodness-of-fit giving a Pearson of 0.49, showing that BMI was related to psychiatric morbidity. In Table 6, depression was related duration on ARV medication and the viral load.

**TABLE 5:** Relationship between clinical characteristics and psychiatric comorbidity.

Clinical parameters	Psychiatric comorbidity						χ^2^	*P*
	Absent (*N* = 56)		Present (*N* = 272)		Total (*N* = 328)			
	*n*	%	*n*	%	*n*	%		
**BMI**							8.64	0.03*
< 18.5	5	12.2	36	87.8	41	12.5	-	-
18.5–24.9	22	12.9	148	87.1	170	51.8	-	-
25–30	21	27.3	56	72.7	77	23.5	-	-
> 30	8	20.0	32	80.0	40	12.2	-	-
Total	56	17.1	272	82.9	328	100.0	-	-
**Adherence**							0.672	0.41
No	20	19.6	82	80.4	102	31.1	-	-
Yes	36	15.9	190	84.1	226	68.9	-	-
Total	56	17.1	272	82.9	328	100.0	-	-
**Frequency hospital visits**							3.12†	0.19
1–2 per year	45	16.2	233	83.8	278	84.8	-	-
3–4 per year	9	28.1	23	71.9	32	9.8	-	-
5–6 per year	2	11.1	16	88.9	18	5.5	-	-
Total	56	17.1	272	82.9	328	100.0	-	-
**Regimen**							3.14	0.08
First line	54	18.3	241	81.7	295	89.9	-	-
Second line	2	6.1	31	93.9	33	10.1	-	-
Total	56	17.1	272	82.9	328	100.0	-	-
**Duration of treatment**							6.08	0.11
≤ 5 years	7	11.3	55	88.7	62	18.9	-	-
6–10 years	9	11.3	71	88.8	80	24.4	-	-
11–15 years	30	21.0	113	79.0	143	43.6	-	-
≥ 16 years	10	23.3	33	76.7	43	13.1	-	-
Total	56	17.1	272	82.9	328	100.0	-	-
**Viral load (copies/mL)**							1.69†	0.43
Undetectable	25	19.7	102	80.3	127	38.7	-	-
≤ 1000	31	16.2	160	83.8	191	58.2	-	-
> 1000 to < 5000	0	0.0	3	100.0	3	0.9	-	-
≥ 5000	0	0.0	7	100.0	7	2.1	-	-
Total	56	17.1	272	82.9	328	100.0	-	-
**WHO HIV stage**							0.72†	0.89
One	53	17.3	254	82.7	307	93.6	-	-
Two	3	16.7	15	83.3	18	5.5	-	-
Three	0	0.0	2	100.0	2	0.6	-	-
Four	0	0.0	1	100.0	1	0.3	-	-
Total	56	17.1	272	82.9	328	100.0	-	-

BMI, body mass index; HIV, human immunodeficiency virus; WHO, World Health Organization.

† Fisher’s exact test used when cell counts < 5.

* , significant *p* < 0.05.

**TABLE 6:** Relationship between clinical parameters and psychiatric morbidity.

Clinical parameters	Depression	Suicide	Mania	Psychosis	Social phobia	PTSD
**Duration on antiretrovirals**						
Pearson chi-square	8.63	2.34	6.67	1.32	4.07	1.10
df	3	3	3	3	3	3
*P*	0.03*	0.51	0.08	0.72	0.25	0.78
**Current line of management**						
Pearson chi-square	2.77	0.87	0.05	1.67	0.17	0.001
df	1	1	1	1	1	1
*P*	0.09	0.35	0.82	0.20	0.68	0.98
**Adherence**						
Pearson chi-square	0.32	0.262	0.98	4.89	1.83	1.73
df	1	1	1	1	1	1
*P*	0.57	0.61	0.32	0.03*	0.18	0.19
**Viral load suppression achieved**						
Pearson chi-square	1.16	1.03	0.971	1.32	0.53	0.25
df	1	1	1	1	1	1
*P*	0.28	0.31	0.32	0.25	0.47	0.06
**WHO HIV and AIDS stage**						
Pearson chi-square	4.68	10.38	3.46	0.88	1.95	1.37
df	3	3	3	3	3	3
*P*	0.20	0.02*	0.33	0.83	0.58	0.71
**Frequency of hospital visits per year**						
Pearson chi-square	5.16	0.21	4.76	0.97	3.29	0.17
df	2	2	2	2	2	2
*P*	0.08	0.09	0.09	0.61	0.19	0.92
**Viral load**						
Pearson chi-square	15.84	2.69	1.26	1.18	1.74	3.03
df	3	3	3	3	3	3
*P*	0.001**	0.44	0.73	0.76	0.63	0.39

PTSD, post-traumatic stress disorder; HIV, human immunodeficiency virus; AIDS, acquired immunodeficiency syndrome; WHO, World Health Organization.

* , *p* < 0.05;

** , *p* < 0.01.

## Discussion

Human immunodeficiency virus is associated with significant unrecognised psychiatric comorbidity. This may be because the distress associated with a major mental disorder is subsumed under the distress associated with the HIV infection itself. In other cases, physicians do not assess for the psychological disorders, thus missing opportunities to help the patient. The prevalence of psychiatric comorbidity is higher in this study (82.9%) and differs from the prevalence in other African studies 71.4% and 45% respectively in this study (82.9%), differing from other African studies with 71.4% and 45%, respectively,^1,5^ and Nigerian studies 48%.^6^ Different factors may account for this, including the fact that different instruments were used. The Nigerian study assessed mainly for depression and anxiety. Respondents in those studies also came from different socioeconomic backgrounds. In addition, there is a long running insurgency in surrounding states with almost two-thirds of residents of some states witnessing severe ethnoreligious violence and 46.1% of respondents in some areas developing PTSD,^21^ while in internally displaced camps, more than three quarters of respondents (78.0%) may have PTSD.^22^ When these factors are coupled with the current socioeconomic status of many respondents, it may account for the higher prevalence. However, this higher prevalence might also be due to various neurocognitive factors, which were not assessed for, such as central nervous system (CNS) toxicity of ARVs^23^ and viral escape.^24^

Psychiatric comorbidity was higher in females. Studies have corroborated the higher incidence of psychiatric comorbidity in women with HIV,^1,25^ usually from depression and anxiety disorders. This might have been due to the fact that there were more females in these studies. However, it could also be due to treatment seeking behaviour among women,^26^ who access HIV testing services more than men^26^ and tend to seek for healthcare earlier. Women are introduced to formal healthcare more readily during pregnancy, labour, immunisations and childcare and so have a better chance of psychiatric comorbidity being recognised while men may present later. In addition, being female is associated with higher odds of having detectable viral load,^27^ which is associated with depression in this study.

The family was still the bastion of care with more than 90% of respondents living within the family circle. However, four-fifths of those who lived alone were females. In such a strongly patriarchal society, marriage is usually the major reason why women move away from parent’s homes. A high proportion of females living alone could thus be due to the presence of discrimination and stigma, the experience of which could be contributory to the presence of psychiatric comorbidity.

Psychiatric comorbidity was unrelated to drugs of abuse both licit and illicit, even though the prevalence of use of these substances was high, consistent with Nigerian studies in PLWHA.^6^ This may be due to the widespread availability of these substances and the use of these substances as a maladaptive coping mechanism for the stress related to living with a chronic illness or a means to ameliorate symptoms of psychological distress.

About a quarter of respondents had experienced psychotic symptoms in the last month. Nigerian studies have shown that almost two-thirds of non-clinical members of the general population exhibit psychotic-like events with up to 13% of them having clinically significant psychotic-like events.^28^ This discrepancy between clinical and non-clinical samples highlights the gap between those who need evaluation and those who present for treatment. In addition, people may experience psychotic symptoms without significant distress and it may go unrecognised. Also, because of cultural factors, people may hold a general belief that people are trying to harm them especially if they have a serious medical disorder (since it is believed that these disorders have ‘spiritual causation’), which is why patients often visit native healers or ‘spiritual healers’ before coming to the hospital. However, these beliefs even though strongly held are not considered abnormal as other members of the socioethnic group also hold on to the same belief.

The prevalence of depressive disorder among PLWHA in studies is quite variable. In the United States, it can be up to 30% – 40%,^29^ in African studies up to 32.2%^1^ and in Nigerian studies 39.6%.^25^ Depressive disorders have been shown to exist with chronic medical conditions and may be the primary cause of previously unrecognised psychiatric comorbidity.^1^ People living with HIV and AIDS may become depressed as a reaction to the illness and go through the stages of grief.^2^ The depressed mood in HIV may be linked to neuroinflammation and stress-induced neuroendocrine changes among other causes.^3^

While having thoughts of suicide is not a disorder, suicide ideation often occurs in depressive disorders and is linked to suicide acts.^25^ However, suicide ideation can occur without the presence of depression.^30^

Human immunodeficiency virus and acquired immunodeficiency syndrome is highly stigmatised despite the many social media campaigns against it. This may account for why social phobia and the fear of being identified or being the focus of attention was observed in more than two-thirds of respondents, as they may be experiencing internalised stigma.

The prevalence for manic or hypomanic symptoms was higher than other studies.^31^ However, this study did not differentiate between the two states. In assessing for mania, irritability is one of the major symptoms. Studies on ‘psychological reactance’ in women with HIV have shown increased irritability due to stigma and discrimination.^32^ The limitations PLWHA place on themselves but which they are unhappy about can manifest through ego defence mechanisms as irritability. In addition, irritability is a common symptom, especially in older PLWHA, which may herald neurologic dysfunction especially in older adults.^33^ At first glance, a subset of patients exhibiting depressive and manic or hypomanic symptoms simultaneously might seem odd. However, bipolar disorders are disorders of dimensions of mood and can present at any point between the dimensions.

Respondents who were not adherent to HIV medication were more likely to have psychotic features. Patients who are not adherent with HIV medication are also unlikely to be adherent to antipsychotics and are thus likely to be more symptomatic especially when they lack ‘insight’.

Adherence was unrelated to social phobia and coming for follow-up. This could be a result of the effectiveness of the campaigns against stigmatising PLWHA and counselling they receive at follow-up. In addition, patients may have seen the improvements in quality of life since they started medication with a sort of positive feedback mechanism, coupled with the fact that PLWHA groups now exist where there is little fear of not being accepted.

Viral load was related to depression. The explanation for this is probably multifactorial with patients with higher viral loads being relatively ‘sicker’ and having more AIDS defining disorders. The frequency of hospital visits was not related to psychopathology except panic disorder. Generally, patients are given shorter appointments when they require close monitoring. More frequent hospital visits decrease the chance of missing symptoms which may be of clinical importance. However, the patient may perceive frequency to mean the HIV treatment is not working and this may lead to anxiety and panic especially when clinic appointments are imminent while awaiting the result of the latest viral load.

Most patients were on first-line medication, and those on second-line medication were more likely to develop a psychiatric comorbidity. This might lend credence to the antiretroviral toxicity theory.^23^

Duration on highly active anti retro-viral therapy (HAART) was related to depression and just five (1.5%) of the patients were in stage three or four despite having been on medication for more than 10 years, testament to the effectiveness of antiretrovirals. Human immunodeficiency virus stage was unrelated to psychiatric disorders screened for but was related to having suicide ideation which could be due to the chronicity of the illness.

### Strengths of the study

Apart from contributing to the body of literature on psychiatric morbidity, one of the advantages of this study was that it was able to identify patients who had previously unrecognised mental illness, as well as showing that social phobia and its attendant avoidance behaviour is still prevalent. This is in addition to showing the importance of adherence to antiretrovirals in the prevention of psychiatric morbidity. Finally, this study underscores the need for integrating mental healthcare into HIV treatment and prevention services.

### Limitations

This was a hospital-based study in a tertiary referral centre with a limited population and as such is likely to overestimate the prevalence of conditions studied and it may be difficult to generalise the results of this study to other settings. This was also a cross-sectional study and attribution is difficult to make. In addition, the model used to determine ‘psychiatric comorbidity’ may overestimate the prevalence of psychiatric comorbidity, as in clinical practice, the best fit diagnosis would be given. Also, ‘false positives’ can be quite high when using instruments. The same patient may meet multiple psychiatric diagnostic criteria, but in clinical practice, the best fit diagnosis would be used as the primary diagnosis to be managed. Finally, neurocognitive symptoms which could be the bridge between HIV infection and the development of psychiatric disorders were not explored.

### Recommendation

This study showed a high prevalence of undetected psychiatry disorders among HIV patients. It is, therefore, recommend that these patients are frequently assessed to facilitate early detection and treatment of identified psychiatry comorbidities. This will subsequently impact on the HIV treatment outcome.

## Conclusions

People living with HIV and AIDS present with substantial psychiatric comorbidity, which is often unrecognised. The most common disorders are social phobia and mixed depression anxiety disorders. Patients who are non-adherent and those on second-line medication were more likely to have psychiatric morbidity. Thus, patients need to be screened for psychiatric comorbidity and managed appropriately when they present for their medication refills in addition to the counselling on medication adherence.
